# Meaning-making while staying connected matters in psychological adaptation during pandemic: a longitudinal moderated mediation study

**DOI:** 10.3389/fpsyg.2024.1364903

**Published:** 2024-02-29

**Authors:** Bin-Na Kim, Hyo Shin Kang, Jungkyu Park

**Affiliations:** ^1^Department of Psychology, Gachon University, Seongnam, Republic of Korea; ^2^Department of Psychology, Kyungpook National University, Daegu, Republic of Korea

**Keywords:** psychological distress, posttraumatic growth, social support, event-related rumination, pandemic

## Abstract

Adversity may bring about both negative and positive changes in psychological adaptation. Although there is mounting evidence regarding the psychological distress during the pandemic, the other side of posttraumatic change, posttraumatic growth (PTG) and its predictors are relatively underexamined. Moreover, there is a paucity of longitudinal investigations that examined intra- and interpersonal predictors responsible for both sides of psychological adaptation. Therefore, this study comprehensively examined the longitudinal relationship among cognitive processing, social support, and adaptation during the pandemic using a moderated mediation model. Specifically, it was tested whether two types of event-related rumination mediated the link between perceived stress and ambilateral adaptational outcomes, and whether social support moderated the mediating pathways of ruminations on adaptation. After informed consent, a representative sample of adults was followed up for over a year, and answered a package of online questionnaires. The results showed that intrusive rumination prospectively predicted greater psychological distress and less PTG in response to stress, whereas deliberate rumination led to less psychological distress and more PTG over time. As predicted, the indirect protective effect of deliberate rumination was stronger when perceived social support was higher. This longitudinal study highlighted the core factors responsible for continued suffering and personal growth during the pandemic. These results have both practical and clinical implications for mental healthcare in the post-COVID era, when the heterogeneity of psychological adaptation increases and preparation for the next pandemic is warranted.

## 1 Introduction

The Chinese word for “crisis” is made up of two seemingly-opposite characters signifying “danger” and “opportunity.” This is also true for human psychological responses to adversity. Traumatic or highly stressful life events may lead to negative changes that can manifest as mental disorders. In contrast, research indicated that certain people may also experience posttraumatic growth (PTG), which is positive changes arising as a result of struggling with trauma, including changed self-perception, relationship with others, and philosophy of life (Tedeschi and Calhoun, 1996). Such bidirectional impact of trauma on human adaptation has been consistently reported in various cases of natural (e.g., earthquake, tsunami) and social (e.g., war, mass shooting) disasters (Orcutt et al., 2014; García et al., 2015; Tsai et al., 2016; Michélsen et al., 2017), although these negative and positive changes are not on opposite poles of one dimension and coexist in an individual level (Taku et al., 2021).

The most recent global crisis was caused by the coronavirus disease 2019 (COVID-19). Even though the acute phase of fear and uncertainty subsided, experts argued that the world must be ready to respond to the next pandemic based on lessons learned from the COVID-19 outbreak (Sachs et al., 2022). COVID-19 was a chronic and complex disaster that entailed both biomedical threat and social disruption. Therefore, a great number of studies have been instantly launched to demonstrate an increase in stress and mental health problems, such as anxiety, depression, and even posttraumatic stress disorder (PTSD) since the beginning of the pandemic (Xiong et al., 2020 for a review). Relatively, the other side of the posttraumatic change, i.e., the PTG during the pandemic was underexamined during the initial period, but studies soon began to capture the phenomenon and predictors of PTG during the COVID-19 crisis (Cui et al., 2021; Hyun et al., 2021; Shigemoto, 2022).

Cognitive processing of traumatic events plays a crucial role in explaining different trajectories of psychological adaptation. Rumination is a transdiagnostic mediator that underlies the link between stress and various adaptational outcomes by intensifying or maintaining stress responses (Hsu et al., 2015). More specifically, two distinct subtypes of event-related rumination have been proposed to be relevant in the aftermath of traumatic events (Cann et al., 2011). Intrusive rumination (IR) refers to the involuntary invasion of negative, repetitive thoughts, whereas deliberate rumination (DR) is defined as more effortful and meaning-making thoughts. Although there is a considerable positive correlation between these two types of event-related rumination, it is intriguing that they possess differential implication for subsequent adaptation. It is well-replicated that IR exacerbates psychological distress in response to stressful or traumatic events; however, its role in PTG remained less clear (Taku et al., 2009, 2021; Cann et al., 2010; Xu et al., 2019). By contrast, a positive relationship between DR and PTG has been fairly consistently reported (Taku et al., 2009; Zhou et al., 2015; Xu et al., 2019). However, it remains unclear whether DR prevents or ameliorates psychological distress (Zhou et al., 2015).

With regards to COVID-19, a similar pattern of differential relationship between two types of event-related rumination and adaptational outcomes also emerged (Cui et al., 2021; Hyun et al., 2021; Ikizer et al., 2021; Kang and Kim, 2021; Zeng et al., 2021; Wall et al., 2022; Xie et al., 2022). Overall, these studies indicate that the extent to which individuals engage in IR and/or DR could explain individual differences in adaptation during a pandemic, as in other cases of adversity. Moreover, Squires et al. (2022) recently reported that IR partially mediated the association between pandemic stress and both depression and anxiety severity, but DR did not. However, few studies have considered event-related ruminations as a mediating mechanism through which individuals’ responses to a pandemic translate into both negative and positive consequences. Moreover, as most of the existing studies were cross-sectional, it was difficult to ascertain the directionality between event-related rumination and adaptational outcomes.

Social support is another factor that should be considered in conjunction with rumination. Numerous studies have confirmed the benefits of supportive social bonds in protecting mental health in the face of adversity (Saltzman et al., 2020). In contrast to other adversities, COVID-19 created a unique situation in which the need for social support increased, but the chance for direct interpersonal contact dramatically decreased because of the social distancing policy implemented globally to prevent the spread of the infection.

Previous studies showed that maintaining SS was associated less psychological distress and greater PTG level during the pandemic (Hyun et al., 2021; Kang and Kim, 2021; Matos et al., 2021). Although SS can buffer psychological distress and contribute to PTG in itself, it is also plausible that the level of perceived SS would moderate the mediating effect of rumination on adaptation, as was the case in a handful of cross-sectional studies that showed that the negative influence of rumination was weakened in participants with higher SS during COVID-19 (Ye et al., 2020; He et al., 2021). In particular, we expected the moderating effect of SS to be more prominent in the mediating pathways of DR, as self-disclosure and positive responses from others after the disclosure, adjacent to what is achieved through perceived SS, were considered highly important in fostering PTG (Park et al., 1996; Tedeschi and Calhoun, 2004).

Building on the theoretical frameworks of rumination, social support, and PTG, this study comprehensively investigated the longitudinal relationships between cognitive processing, SS, and adaptation during the pandemic using a moderated mediation path model. It was examined whether two types of event-related rumination, IR and DR, would mediate the link between perceived stress and bilateral adaptational outcomes, psychological distress, and PTG in a representative sample of Korean adults who were followed-up over a one-year interval during the COVID-19 crisis. Furthermore, it was tested whether social support would moderate the mediation of ruminations on adaptation. More specifically, it was expected that the indirect effect of DR between perceived stress and PTG would be stronger in participants with higher SS.

*Hypothesis 1*: Two types of event-related rumination would mediate the link between perceived stress and bilateral adaptation outcomes, psychological distress and PTG.

*Hypothesis 2*: SS would moderate the mediation of rumination on adaptation. In particular, the indirect effect of DR between perceived stress and PTG would be stronger in participants with higher SS.

## 2 Methods

### 2.1 Participants and procedures

Data were collected as part of a longitudinal study on psychological adaptation during the pandemic. The procedures and materials were approved by the Institutional Review Board of Kyungpook National University (KNU-2020-0054/KNU-2021-0119). Time 1(T1) data were collected in August 2020 from the greater Daegu area, where the first massive outbreak occurred in South Korea and residents experienced widespread infection with dread in the acute phase. Through an online panel research company, we recruited a representative sample of adults (20 years or older) in terms of age and gender to enhance the generalizability. After informed consent, 316 adults (*M* = 43.27 years, *SD* = 12.61, 50.6% female) completed an online questionnaire that included a variety of measures relevant to psychological adaptation during the pandemic at T1 (Table A1). After a year, Time 2 (T2) data were collected in August 2021, when Delta became the predominant variant leading to an overwhelming increase in infection and hospitalization nationwide. In the second assessment, 141 adults (*M* = 49.14, *SD* = 10.57, 44.6% female), which constituted 48.4% of the original participants, completed an online follow-up survey. To investigate the potential impact of selective attrition, we tested the differences in the main variables in the first assessment between the retained and drop-out participants, and found that there was no significant difference except for age (*t*(314) = −3.89, *p* < 0.001).

### 2.2 Measures

#### 2.2.1 Demographics and COVID-19-related variables

The participants were asked to report their age, gender, educational level, and marital status. Questions regarding COVID-19-related experiences were also included. In addition to questioning whether they had experienced a COVID-19 diagnosis (themselves, family members, or close friends), screening tests, self-quarantine, or vaccination, two five-point Likert scales (from 1 = not at all to 5 = very much) were included to measure the subjective severity of COVID-19-related experiences (disruption in daily life *M* = 3.67, *SD* = 0.82 and perceived traumatic experience *M* = 2.75, SD = 1.01), adapted from García et al. (2015).^1^

#### 2.2.2 Korean version of the perceived stress scale (K-PSS)

The PSS was devised to assess the degree of individual’s perceived stress in daily life (Cohen et al., 1983). The K-PSS was used in this study (Lee et al., 2012), which consists of 10 items rated on a 5-point Likert scale (from 0 = never to 5 = very often). The internal consistency was adequate in this study (Cronbach’s *α* = 0.76).

#### 2.2.3 Korean version of the brief symptom inventory (K-BSI)

The BSI is a widely used self-report questionnaire that assesses psychological distress ranging from depression, anxiety, and somatization (Derogatis, 2001). It contains 18 items rated on a five-point Likert scale (from 1 = not at all to 5 = very much). The K-BSI was validated by Park et al. (2012). The total score of the three subscales, global severity index (GSI), was used as an index for the general level of psychological distress (Cronbach’s *α* = 0.96).

#### 2.2.4 Korean version of the post-traumatic growth inventory expanded (K-PTGI-X)

The PTGI-X is a revised version to enhance the validity of the spiritual and existential change components of the prior version of the PTGI (Tedeschi et al., 2017). In the Korean version validated by Kim et al. (2020), participants were asked to answer 25 items on a six-point Likert scale (from 1 = I did not experience this change to 6 = I experienced this change to a very great degree), which covers four domains of growth: personal strength (8 items), relating to others (5 items), new possibilities (5 items), and spiritual and existential change (7 items). To focus on assessing the pandemic-related PTG, we specified “changes after you experienced the COVID-19-related situations” in the instructions. The overall internal consistency was excellent (Cronbach’s *α* = 0.97).

#### 2.2.5 Korean version of the event-related rumination inventory (K-ERRI)

The ERRI was developed to assess two types of rumination during major life crises: intrusive and deliberate rumination (Cann et al., 2011). The K-ERRI was used in this study (Ahn et al., 2013). It consists of 20 items (10 items for intrusive and deliberate rumination, respectively) rated on a four-point Likert scale (from 0 = not at all to 3 = often). To specifically capture event-related rumination in response to the pandemic, the researchers slightly changed the wording of the phrase “during the weeks immediately after the event” into “during COVID-19.” Higher score indicated higher levels of intrusive and deliberate rumination. The internal consistencies of both the subscales were excellent in this study (Cronbach’s α: intrusive rumination = 0.96; deliberate rumination = 0.91).

#### 2.2.6 Perceived social support

Perceived social support was assessed by using a self-reporting questionnaire (Park, 1985), which included emotional, material, informational, and evaluative social support (Cronbach’s *α* = 0.98) with 25 items rated on a five-point Likert scale (from 1 = not at all to 5 = very much).

### 2.3 Statistical analysis

In the path analysis, the hypothesized model posited that the relationships between perceived stress at T1 (PSS-1) and the two outcome variables, psychological distress at T2 (BSI-2) and posttraumatic growth at T2 (PTG-2), were mediated by two mediators — intrusive rumination at T1 (IR-1) and deliberate rumination at T1 (DR-1). Moreover, social support at T1 (SS-1) was thought to moderate the relationships between PSS1 and its two mediators. The parameters estimated in the study model were utilized to estimate the indirect effects, each mediated by one of the mediators to each outcome variable, moderation effects, and moderated mediation effects.

To test for indirect effects, 95% CIs for the average indirect effects were constructed using Monte Carlo simulation procedure. This approach reflects the asymmetric nature of the sampling distribution of an indirect effect accurately by producing empirical sampling distributions of the path coefficients used to calculate the indirect effect (Preacher and Selig, 2012).

The significance of the moderating effect was evaluated using a simple slope test (Aiken and West, 1991; Preacher et al., 2006), whereas the moderated mediation effect was examined following the guideline by Edwards and Lambert (2007). This method evaluates whether the indirect effect varies at high and low levels of SS-1 using indirect effects derived from sampling distributions. We generated 50,000 sets of parameters estimates and constructed 95% CIs for the indirect and moderated mediation effects. All statistical analyses were conducted using Mplus version 8.6 (Muthén and Muthén, 1998–2017).

## 3 Results

### 3.1 Indirect effects of event-related rumination

**Figure 1:**
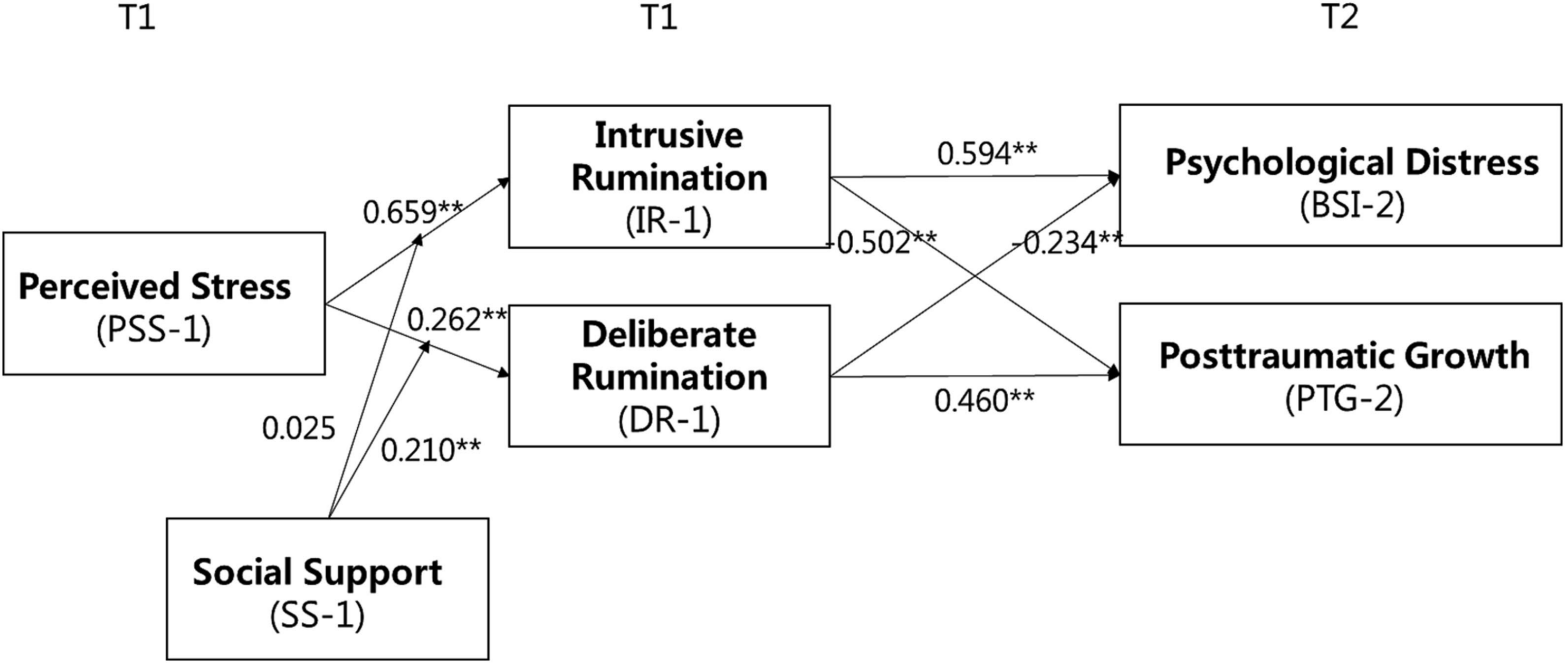
Parameter estimates in the hypothesized path model. Controlled for age, gender, and subjective severity of COVID-19-related experiences. **p < 0.01.

Figure 1 displayed the estimated parameters of the hypothesized path model. These parameter estimates were used to compute the indirect, moderation, and moderated mediation effects. As can be seen in Table 1, PSS-1 and BSI-2 were positively related through IR-1 (estimate = 1.27, 95% CI [0.830, −0.907]), whereas PSS-1 and PTG-2 revealed a negative relationship through the same mediator (estimate = −1.50, 95% CI [−2.200, −0.907]). On the other hand, PSS-1 was negatively associated with BSI-2 via DR-1 (estimate = −0.19, 95% CI [−0.457, −0.024]), while PSS-1 and BSI-2 showed a positive relationship through the same mediator, DR-1 (estimate = 0.53, 95% CI [0.151, 1.048]). These results provided support for Hypothesis 1.

**Table 1:** Parameter estimates of indirect effects.

Mediating pathway	*β*	SE	*p*	95% C.I.
(1) PSS-1➔IR-1➔BSI-2	1.27	0.25	<0.001	[0.830, 1.778]
(2) PSS-1➔IR-1➔PTGI-2	−1.50	0.33	<0.001	[−2.200, −0.907]
(3) PSS-1➔DR-1➔ BSI-2	−0.19	0.11	<0.001	[−0.457, −0.024]
(4) PSS-1➔DR-1➔ PTGI-2	0.53	0.23	<0.001	[0.151, 1.048]

PSS, Perceived Stress Scale; IR, intrusive rumination subscale of the Event-Related Rumination Inventory; DR, deliberate rumination subscale of the Event-Related Rumination Inventory; PTGI, Post-traumatic Growth Inventory Expanded.

### 3.2 Moderation effects of social support

**Figure 2:**
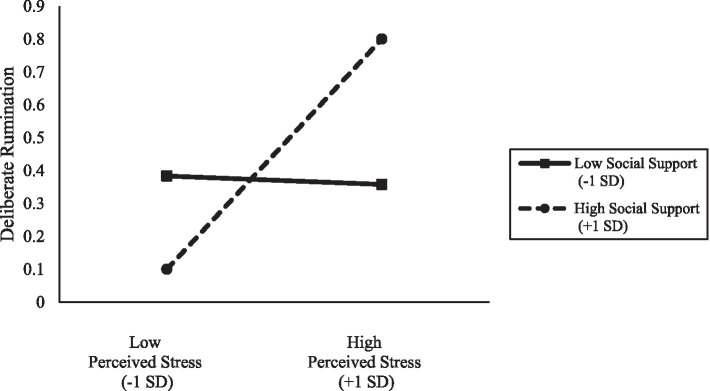
The moderation effects of social support on deliberate rumination.

Figure 1 also showed the relationship between PSS-1 and DR-1 was moderated by SS-1 (*γ* = 0.210, *p* < 0.01); however, the moderation effect of SS-1 on the relationship between PSS-1 and IR-1 was not significant (*γ* = 0.025, ns). As shown in Figure 2, the simple slope test indicated that the moderating relationship between PSS-1 and DR-1 was stronger for whose social support level was high (*B* = 0.40, *p* < 0.01) versus low (*B* = −0.01, ns).

### 3.3 Moderated mediation effects of social support

Table 2 indicated that the indirect effect of PSS-1 and BSI-2 through DR-1 was significant under conditions of both higher (estimate = −0.24, 95% CI [−0.511, −0.046]) and lower social support (estimate = −0.18, 95% CI [−0.451, −0.022]). Difference between these two indirect effects under different levels of social support was found to be also significant (estimate = −0.05, 95% CI [−0.126, −0.002]). These results showed that the indirect effect of PSS-1 on BSI-2 via DR-1 was stronger among participants with higher levels of social support than among those with lower levels.

**Table 2:** Parameter estimates of indirect effects under different levels of social support.

Mediating pathway	*β*	SE	95% C.I.
(1) IE for high SS-1 (PSS-1➔ DR-1➔ BSI-2)	−0.24	0.12	[−0.511, −0.046]
(2) IE for Low SS-1 (PSS-1➔ DR-1➔ BSI-2)	−0.18	0.11	[−0.451, −0.022]
(3) Difference in (1) and (2)	−0.05	0.03	[−0.126, −0.002]
(4) IE for high SS-1 (PSS-1➔ DR-1➔ PTGI-2	0.67	0.22	[0.297, 1.167]
(5) IE for Low SS-1 (PSS-1➔ DR-1➔ PTGI-2)	0.53	0.23	[0.139, 1.034]
(6) Difference in (4) and (5)	0.14	0.07	[0.018, 0.297]

IE, Indirect effect; SS, Perceived Social Support Scale; PSS, Perceived Stress Scale; IR, intrusive rumination subscale of the Event-Related Rumination Inventory; DR, deliberate rumination subscale of the Event-Related Rumination Inventory; PTGI, Post-traumatic Growth Inventory Expanded.

Moreover, as shown in Table 2, the indirect effects of PSS-1 and PTG-I through DR-1 were significant under conditions of both high (estimate = 0.67, 95% CI [0.297, 1.167]) and low social support (estimate = 0.53, 95% CI [0.139, 1.034]). Notably, the difference between the two indirect effects was significant (estimate = 0.14, 95% CI [0.018, 0.297]). In support of Hypothesis 2, these findings suggested that the indirect effect was more prominent in participants with higher levels of social support in comparison to those with lower levels.

## 4 Discussion

Consistent with previous studies within and outside the COVID-19 context, the current study replicated the finding that IR aggravates psychological distress, whereas DR promotes PTG during highly stressful times. However, in contrast to other cross-sectional studies, we demonstrated that both IR and DR can predict bidirectional adaptational consequences over time. Our results showed that the indirect paths of IR and DR between perceived stress and ambilateral adaptational outcomes were all significant. That is, engaging in IR toward increased stress in the acute phase of the pandemic led to higher levels of psychological distress and lower levels of PTG 1 year later. Meanwhile, DR prospectively predicted higher levels of PTG and lower levels of psychological distress. Despite the substantial positive correlation between these two types of event-related rumination (Zhou and Wu, 2016; Kang and Kim, 2021), IR and DR were differentially related to psychological adaptation.

In particular, it is worth noting that this study overcame certain limitations of previous research that was cross-sectionally designed, dealt with only one aspect of adaptation, or used convenience student samples. Longitudinal investigations of the differential mediating effects of IR and DR in both negative and positive consequences are sparse to date, and even fewer in the COVID-19 context (Squires et al., 2022). For instance, a longitudinal study by Hyun et al. (2021) explored psychosocial correlates (e. g., demographics, psychiatric symptoms, and COVID-19-related worry) of PTG only, but did not considered event-related rumination as mediator or psychological distress as outcome. However, a few studies have shown that different cognitive processing of traumatic events, such as earthquakes, could explain individual differences in adaptation over time. For example, Zhou and Wu (2016) demonstrated that IR led to PTSD symptoms, whereas DR elicited PTG at 18 months after the Ya’an earthquake in Chinese adolescents. But, to date, no study longitudinally investigated the whole picture at the intersection of rumination, social support, and both sides of adaptation during the COVID-19. By utilizing two-wave prospective design, the temporal precedence of event-related rumination was specified and possible causal relations between cognitive processing and subsequent adaptation could be clarified, indicating that *how* people attend to their stressful experiences is a potent mediator of final adaptational outcomes. In addition, the use of a representative sample enabled us to interpret these results with enhanced generalizability.

Furthermore, a more interesting picture emerged from the moderated mediation analysis. While the indirect effects through IR did not significantly differ depending on perceived social support was high or low, differences in the moderated mediation effect through DR were all significant. Consistent with our expectations, the indirect effect of DR between perceived stress and PTG was stronger when perceived social support was higher. Conversely, the indirect effect of DR between stress and psychological distress became weaker when perceived social support was higher. Taken together, these results indicate that the beneficial effect of DR is more contingent on the level of social support than the detrimental effect of IR. When the longitudinal relationship was examined, considering perceived social support as a moderator, the protective role of DR became more apparent. When the distinction between the search for meaning and the presence of meaning is considered (Park, 2010; Shin and Steger, 2016), this result could be because searching for meaning after adversity could occasionally be painful or even unsuccessful, and moving forward to the meaning-making process could be effortful, especially in the absence of continued social support. In this regard, supportive responses from others, which provide a kind of positive validation of how they coped with what happened, are considered essential for facilitating PTG (Tedeschi and Calhoun, 2004).

Our results have practical and clinical implications for mental healthcare in the post-COVID era. The present is the time to shift the focus toward the heterogeneity of post-pandemic psychological adaptation. Research on past and most recent pandemics has shown that the general level of psychological suffering may gradually decline over time (e.g., adaptation), whereas vulnerable groups may continue to suffer (Mann and Walker, 2022). On the contrary, some may experience personal growth and thrive, rebuilding their life narratives based on suffering.

This study sheds light on the factors that can cause such differences. First, not how much but how individuals think about stressful experiences affects their psychological adaptation trajectories. Guiding individuals to engage in more constructive and effortful cognitive processing of pandemic-related stress should be an important target for psychological education and treatment manuals. Second, socially disconnected persons with heightened IR and diminished DR would represent the most vulnerable group in need of screening for the prolonged psychological aftereffects of the pandemic. Social disconnection has been increasingly recognized as a “global behavioral epidemic,” and this concern has been exacerbated by the COVID-19 pandemic (Na et al., 2023). The message from our study can be summarized as meaning-making while staying connected is conducive to both alleviating psychological distress and fostering psychological maturation, which is an important lesson for future pandemics.

Several limitations of this study should be considered when interpreting the results. First, although longitudinal, the time frame was not sufficiently long to cover the latter phases of the pandemic. Therefore, it was difficult to ascertain prolonged effects over an extended period of time. Second, only Korean participants were recruited, which may limit the generalizability of our findings. However, this can also be considered a strength, as most previous research has been conducted in the United States, Europe, and China. Third, self-report measures were used. Although few alternative, well-validated measures of cognitive processing, social support are available, diversifying assessment methods including physiological or societal-level measures would be helpful in elucidating the complex, multi-level phenomenon of adaptation.

## 5 Conclusion

In conclusion, this study offers meaningful lessons that would be helpful in preparing for future pandemics by identifying who would have withered or thrived through the most recent pandemic. From a psychological perspective, it is highly important to have a balanced view of the diverse adaptation trajectories during the pandemic, as humans possess the capacity to cope with even the most extreme adversity (Bonanno, 2004). It is hoped that this study can contribute to a better understanding of how people can protect their mental health and mature during and beyond inevitable adversities, including pandemics.

## Data availability statement

The original contributions presented in the study are included in the article/supplementary material, further inquiries can be directed to the corresponding authors.

## Ethics statement

The studies involving humans were approved by Institutional Review Board of Kyungpook National University (KNU-2020-0054/KNU-2021-0119). The studies were conducted in accordance with the local legislation and institutional requirements. The participants provided their written informed consent to participate in this study.

## Author contributions

B-NK: Conceptualization, Funding acquisition, Investigation, Writing – original draft, Writing – review & editing. HK: Conceptualization, Data curation, Investigation, Writing – review & editing. JP: Conceptualization, Formal analysis, Methodology, Writing – original draft.

## Footnotes

^1^The items were as follows. (1) Disruption in daily life: To what degree, do you feel your life was disrupted as a result of COVID-19? (2) Traumatic experience: To what degree, do you rate COVID-19 as a traumatic experience to your life?

## Appendix

**Table A1:** Participants characteristics (Time 1 *N* = 316; Time 2 *N* = 141).

		Mean	SD	N (%)	Mean	SD	N (%)
Age		43.27	12.61		49.14	10.57	
Gender	Male			156 (49.4%)			81 (57.4%)
	Female			160 (50.6%)			60 (42.6%)
Residence	Daegu			220 (69.6%)			96 (68.1%)
	Gyeonsangbuk-do			96 (30.4%)			45 (31.9%)
COVID-19-related experience	Diagnosis confirmed			1 (.3%)			2 (1.4%)
	Diagnosis of family or close friends			31 (9.8%)			11 (7.8%)
	Screening test			42 (13.3%)			16 (11.3%)
	Self-quarantine			23 (7.3%)			13 (9.2%)
Education	University			239 (75.7%)			112 (79.4%)
	High school			74 (23.4%)			28 (19.9%)
	Middle school			3 (.9%)			1 (0.7%)
Marital status	Unmarried			117 (37.0%)			34 (24.1%)
	Married or cohabiting			175 (55.4%)			92 (65.2%)
	Divorced			20 (6.3%)			12 (8.5%)
	Bereaved			4 (1.3%)			3 (2.1%)
